# Photo-catalytic activity of Zn_1-*x*_Mn_*x*_S nanocrystals synthesized by wet chemical technique

**DOI:** 10.1186/1556-276X-6-438

**Published:** 2011-06-24

**Authors:** Mansi Chitkara, Karamjit Singh, Inderjeet Singh Sandhu, Harbhajan Singh Bhatti

**Affiliations:** 1 Nanomaterials Research Laboratory (NRL), Department of Applied Sciences, Chitkara University, Rajpura, Punjab 140 401, India; 2 Department of Physics, Punjabi University, Patiala, Punjab 147 002, India

## Abstract

Polyvinyl pyrrolidone capped Zn_1-*x*_Mn_*x*_S (0 ≤ *x *≤ 0.1) nanocrystals have been synthesized using wet chemical co-precipitation method. Crystallographic and morphological characterization of the synthesized materials have been done using X-ray diffraction and transmission electron microscope. Crystallographic studies show the zinc blende crystals having average crystallite size approx. 3 nm, which is almost similar to the average particle size calculated from electron micrographs. Atomic absorption spectrometer has been used for qualitative and quantitative analysis of synthesized nanomaterials. Photo-catalytic activity has been studied using methylene blue dye as a test contaminant. Energy resolved luminescence spectra have been recorded for the detailed description of radiative and non-radiative recombination mechanisms. Photo-catalytic activity dependence on dopant concentration and luminescence quantum yield has been studied in detail.

## Introduction

Environmental pollution, toxic water pollutants, and industrialization on a global scale have drawn attention for sustained fundamental and applied research in the area of environmental remediation. The increased public concern with environmental pollutants has prompted the need to develop novel treatment methods [1] where photo-catalysis is gaining a lot of attention in the field of pollutant degradation. Semiconductor photo-catalysts offer the potential for complete elimination of toxic chemicals through their efficiency and potentially broad applicability [2,3]. Recently, semiconductor nanocrystals have attracted great attention due to their size tunable physical and chemical properties. Transition from bulk to nanoparticles lead to the display of quantum mechanical properties and an increased dominance of surface atoms, which give rise to unique photo-physical and photo-catalytic properties of nanomaterials, for example, with the decrease of particle size, extremely high surface to volume ratio is obtained leading to an increase in surface specific active sites for chemical reactions and photon absorption to enhance the reaction and absorption efficiency. The enhanced surface to volume ratio causes increase of surface states, which changes the activity of electrons and holes, affecting the chemical reaction dynamics. The size quantization increases the bandgap of photo-catalysts to enhance the redox potential of conduction band electrons and valence band holes [4].

Various new compounds and materials for photo-catalysis have been synthesized in the past few decades [5-13]. Semiconductor photo-catalysts, with a primary focus on TiO_2 _[14-17], have been applied to variety of problems of environmental interest in addition to water and air purification. The application of illuminated semiconductors for degrading undesirable organics dissolved in air or water is well documented and has been successful for a wide variety of compounds [2]. Transition-metal sulphides, in particular ZnS [18,19], have unique catalytic functions as a result of the rapid generation of electron-hole pairs by photo-excitation and the highly negative reduction potentials of excited electrons. Moreover, incorporation of metal ion dopants in these semiconductor nanoparticles can influence their photo-catalytic performance. Doping of Mn^2+ ^ions in ZnS lengthens the lifetime of generated charge carriers, resulting in an enhancement in the photo-activity. Hence, ZnS:Mn^2+ ^nanocrystals can be efficiently used for environmental cleaning, H_2 _production, and water purification. This article reports photo-catalytic activity of Zn_1-*x*_Mn_*x*_S nanocrystals. Photo-catalytic activity has been well correlated with the luminescence quantum yield. Moreover, photo-catalytic and luminescence efficiency dependence on the Mn^2+ ^concentration have been described in detail.

## Experimental

Zn_1-*x*_Mn_*x*_S (0 ≤ *x *≤ 0.1) nanocrystals have been synthesized using wet chemical co-precipitation method already opted by Singh et al. [20] for the synthesis of Eu^3+ ^doped Cd_1-*x*_Zn_*x*_S quantum dots. All synthesis was carried out at room temperature under ambient conditions in aqueous media for its inherent advantages of being simple and environmental friendly. Analytical reagent grade chemicals: zinc acetate (C_4_H_6_O_4_Zn · 2H_2_O), manganese acetate (C_4_H_6_MnO_4 _· 4H_2_O), sodium sulphide (Na_2_S · H_2_O), and polyvinyl pyrrolidone (PVP) [(C_6_H_9_NO)_*n*_] were used without further purification. Solutions of 0.5 M zinc acetate, 0.5 M sodium sulphide, and 1 M manganese acetate were prepared in separate beakers. Then zinc and manganese precursor solutions were mixed in the stoichiometric proportion under vigorous stirring, 4 ml of 2% PVP solution was added to total 50 ml volume, before drop wise addition of sulfur precursor. PVP will act as the capping agent to avoid the agglomeration of nanocrystals. The resulting precipitates were centrifuged and dried in vacuum oven for 10 to 12 h continuously.

Panalytical's (Netherlands) X'Pert Pro Powder X-ray diffractometer with Cu K_α _radiation (λ = 1.541 Å) was used to record diffraction patterns of the synthesized samples in the 2θ range 20 to 60°. Average crystallite size has been calculated from the line broadening of the X-ray diffraction (XRD) diffractogram using Scherrer formula [21]. Hitachi, [(H-7500), Japan] transmission electron microscope (TEM) was used to record micrographs for average particle size determination. For TEM studies, a drop of well ultrasonicated ethanol dispersed nanocrystals was placed on the carbon coated copper grid.

Atomic absorption spectrometer (Analytic Jena, Germany) has been used for qualitative and quantitative analysis of the synthesized nanomaterials. Sample preparation for the analysis involves dissolution of 0.01 mg of nanocrystals in 10 ml of 0.5% HNO_3_.

Energy resolved luminescence spectra were recorded using FlouroMax-3 (Jobin-Yvon, Edison, NJ, USA) spectrofluorometer equipped with photomultiplier tube and a xenon lamp.

The photo-catalytic activity of Zn_1-*x*_Mn_*x*_S nanocrystals was studied by monitoring the degradation of methylene blue (MB) (C_16_H_18_ClN_3_S · 2H_2_O) dye in an aqueous suspension containing Zn_1-*x*_Mn_*x*_S nanocrystals under the UV-radiation exposure with continuous magnetic stirring. A 350 ml of aqueous suspension was prepared by completely dissolving 1.1322 mg of the MB dye and then dispersing 140 mg of the Zn_1-*x*_Mn_*x*_S nanocrystals in the de-ionized water. The resulting suspension was equilibrated by stirring in the dark for 1 h to stabilize the adsorption of MB dye on the surface of nanocrystals. The stable aqueous suspension was then exposed to the UV-radiation with continuous magnetic stirring, using the home made photoreactor containing two 18-W tubes as the UV-source (λ = 200 to 400 nm). Following the UV-radiation exposure, 10 ml sample of aqueous suspension was taken out after every 10-min interval for the total 80 min of the UV-radiation exposure. Suspension sample was centrifuged to filter out the Zn_1-*x*_Mn_*x*_S nanocrystals, then nanocrystal free aqueous dye solution was examined using UV-vis absorption spectrophotometer (Systronics PC Based Double Beam Spectrophotometer:2202) to study the photo-degradation of the MB dye.

## Results and discussion

**Figure 1:**
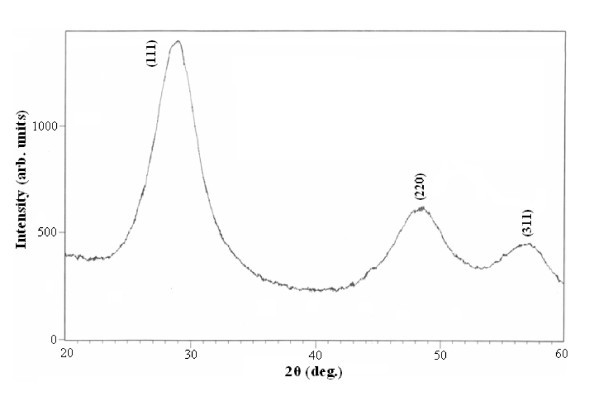
**XRD pattern of Zn**_**0.9900**_**Mn**_**0.0100**_**S nanocrystals**.

Broad XRD patterns have been recorded for all the synthesized Zn_1-*x*_Mn_*x*_S samples, Figure 1 shows one such X-ray diffractogram recorded for Zn_0.9900_Mn_0.0100_S. It is clear from the diffractogram that synthesized samples crystallize in zinc blende structure with the planes at {111}, {220}, and {311}, respectively. Recorded diffraction peaks are broadened due to the nanocrystalline nature of particles. Average crystallite size calculated from the recorded XRD patterns is approx. 3 nm.

**Figure 2:**
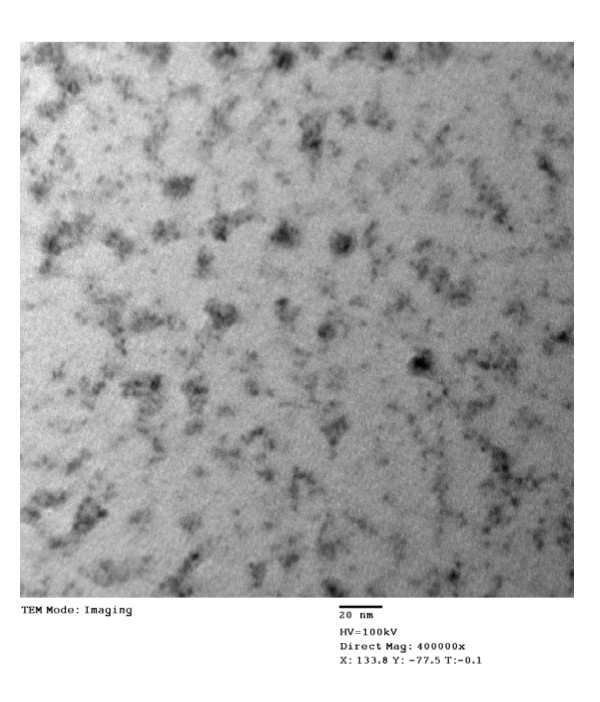
**TEM image of Zn**_**1-*x***_**Mn**_***x***_**S nanocrystals**.

Figure 2 shows the TEM micrograph recorded for Zn_1-*x*_Mn_*x*_S nanocrystals. Average particle size calculated from micrograph is approx. 3 to 4 nm, which is in proximity to the average crystallite size determined by XRD. So, all the synthesized particles are single nanocrystals.

Atomic absorption spectrometer (AAS) studies show that the actual concentration of manganese doping is approx. 24% of the initial manganese precursor concentration, which was added to the reaction media. So, the value of *x *in Zn_1-*x*_Mn_*x*_S corresponds to initial atomic weight concentration of manganese with respect to zinc, which was calculated during stochiometric addition of precursors in the chemical co-precipitation reaction.

**Figure 3:**
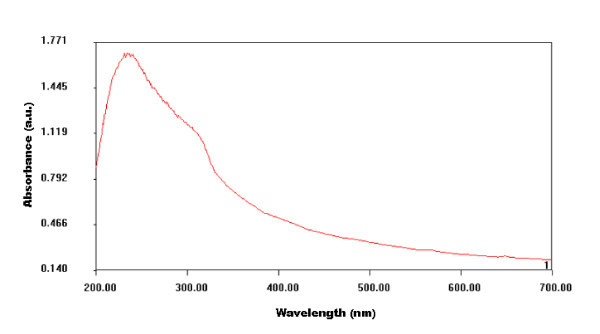
**Absorption spectra of nanocrystals**.

**Figure 4:**
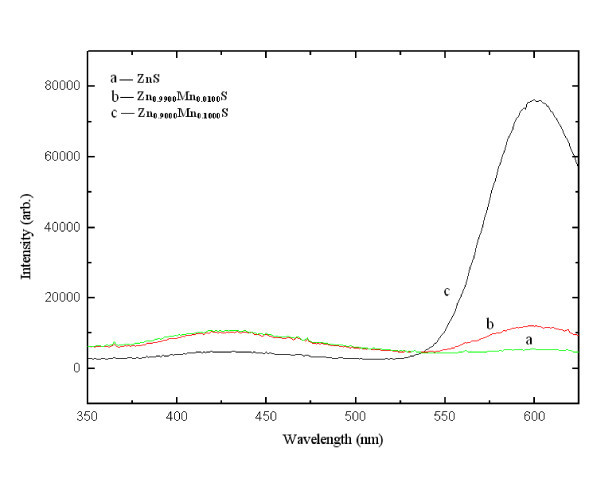
**Photoluminescence spectra of Zn**_**1-*x***_**Mn**_***x***_**S nanocrystals**.

**Figure 5:**
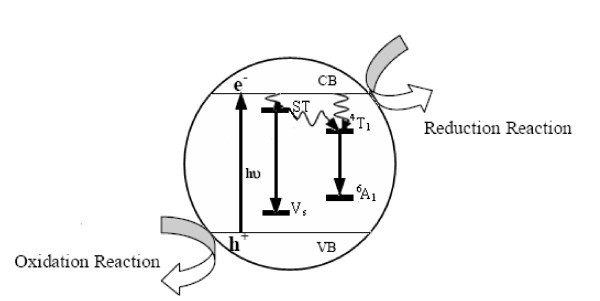
**Processes involved in Zn**_**1-*x***_**Mn**_***x***_**S nanocrystals upon excitation**. CB, conduction band; ST, shallow trap; ^4^T_1 _and ^6^A_1_, manganese levels; VB, valence band; *V*_s_, sulfur vacancy.

Figure 3 shows absorption spectra of synthesized nanocrystals, which lie in UV range. Energy resolved luminescence spectra shown in Figure 4 have been recorded at room temperature using 325-nm excitation. It is clear from the recorded spectrum that pure ZnS nanocrystals show only 425-nm emission peak, whereas dichromatic emission (λ_1 _= 425 nm and λ_2 _= 599 nm) has been observed in case of Mn^2+^-doped ZnS nanocrystals. Luminescence quantum yield of λ_2 _emission peak go on increasing with the increase of '*x*' in Zn_1-*x*_Mn_*x*_S nanocrystals, whereas λ_1 _emission intensity go on decreasing with increasing concentration of Mn^2+ ^ions. More than six-fold increase and two-fold decrease has been observed in the emission intensities of λ_2 _and λ_1 _peaks, respectively, when the value of '*x*' changes from 0.01 to 0.1 in Zn_1-*x*_Mn_*x*_S nanocrystals. The Mn^2+ ^ions substitute the Zn^2+ ^ions in the ZnS nanocrystal acting as trap sites [22], where the electrons and holes can be trapped. Electrons after photo-excitation process in the host lattice subsequently decay via non-radiative process to the ^4^T_1 _localized state of manganese. The λ_2 _(599 nm) emission peak is attributed to the radiative decay between the ^4^T_1 _and ^6^A_1 _localized states of manganese inside the ZnS bandgap. The λ_1 _emission (425 nm) peak is assigned to the radiative transition of electrons from shallow trap states (ST) near the conduction band to sulfur vacancies (*V*_s_) residing near the valence band. The increasing dopant concentration quenches the host related 425 nm emission. Detailed mechanism of various processes involved in Zn_1-*x*_Mn_*x*_S nanocrystals upon excitation is shown in Figure 5. Photo-excited electrons from the conduction band transit spontaneously to the ST and ^4^T_1 _manganese trap sites via non-radiative processes. These ST electrons can recombine radiatively with *V*_s _holes or further relaxed non-radiatively to the localized dopant trapping states. Radiative recombination of ST electrons and *V*_s _holes is faster than the radiative transition between the ^4^T_1 _and ^6^A_1 _localized states [23].

**Figure 6:**
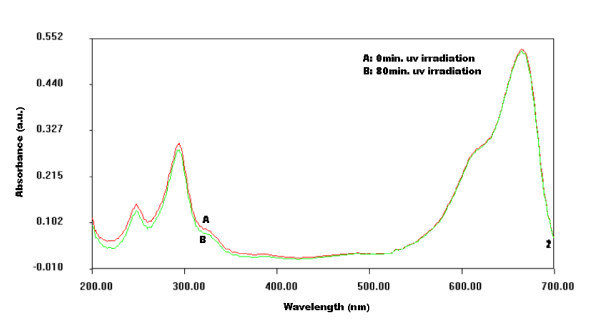
**Absorption spectra of pure MB dye**.

Figure 6 shows absorption spectra of pure MB dye with and without UV radiation exposure. It can be clearly seen from the recorded spectra that both the curves overlap on each other, which confirms that there is no photo-bleaching of pure MB dye.

**Figure 7:**
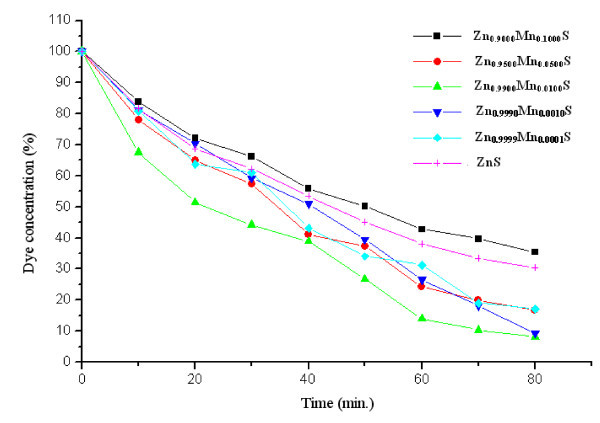
**Photodegradation of MB dye with time**.

**Figure 8:**
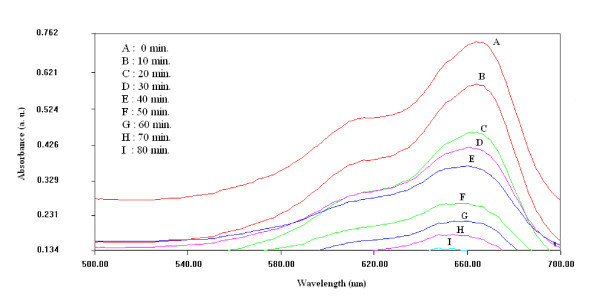
**Absorption spectrum of dye solution for different durations of UV-radiation exposure in the presence of Zn**_**0.9900**_**Mn**_**0.0100**_**S nanocrystals**.

Figure 7 shows the Zn_1-*x*_Mn_*x*_S (0 ≤ *x *≤ 0.1) nanocrystals sensitized photo-degradation of MB dye under UV-radiation exposure. It can be clearly seen that MB dye is degraded to maximum extent in case of Zn_0.9900_Mn_0.0100_S nanocrystals, whereas it is degraded to minimum extent for Zn_0.9000_Mn_0.1000_S nanophoto-catalyst. Photoredox chemistry occurring at nanocrystal surface emanates from trapped charge carriers. Incorporation of Mn^2+ ^in ZnS nanocrystal lattice significantly influences the photo-catalytic activity. Addition of Mn^2+ ^ions lengthens the lifetime of excited charge carriers, which results the enhanced photo-catalytic activity. Various charge carrier recombination and charge carrier trapping processes are shown in Figure 5. The competition between the charge carrier recombination and charge carrier trapping followed by the competition between recombination of trapped carriers and interfacial charge transfer determine the overall quantum efficiency for interfacial charge transfer. Doping of Mn^2+ ^up to optimal concentration increases the interfacial charge transfer probability, due to which photo-catalytic activity of ZnS nanocrystals is enhanced. As shown in Figure 7, photo-catalytic activity enhances with increasing value of '*x*' only in the range *x *= 0 to *x *= 0.01, further increase of dopant concentration, i.e., *x *= 0.01 to *x *= 0.1 deteriorates photo-catalytic activity of Zn_1-*x*_Mn_*x*_S nanocrystals. It is due to the fact that up to optimal Mn^2+ ^concentration (*x *= 0.01), Mn^2+ ^ions lengthens the charge carrier recombination, but at higher dopant concentrations although the possibility of charge carrier trapping is high, but the charge carriers may recombine through quantum tunneling. Moreover, increasing concentration of Mn^2+ ^ions may cause the increased interaction between neighboring Zn^2+ ^ions and the Mn^2+ ^luminescence centre that enhances the spin-orbit coupling of Mn^2+ ^ions, which leads to the relaxation of the spin selection rules [24]. This lowers the radiative recombination time for ^4^T_1 _→^6^A_1 _transitions, so the recombination of trapped carriers dominates interfacial charge transfer at the higher dopant concentrations. Due to enhanced recombination rate luminescence quantum yield increases to large extent as shown in Figure 4. Figure 8 shows the absorption spectrum of MB dye solution for different durations of UV-radiation exposure in the presence of Zn_0.9900_Mn_0.0100_S nanocrystals (optimal dopant concentration). Zn_0.9900_Mn_0.0100_S nanocrystal photo-catalyst is efficiently degrading the dye, only negligible amount of dye is present in the solution after 80 min. There is a concentration dependent slight spectral shift in MB dye absorption spectra as the UV irradiation time changes from 0 to 80 min. Red shift in the absorption peak with increasing dye concentration has been observed due to augmented optical density. Moreover, at higher concentrations, aggregation can take place, which affects the optical behaviour. These non-toxic, stable, inexpensive nanocrystalline photo-catalyst having high-redox potentials can be efficiently used for environmental cleaning, water purification, and H_2 _production. Moreover, due to non-dissolving nature in aqueous media, these photo-catalysts can be easily recovered after use.

## Conclusions

Zn_1-*x*_Mn_*x*_S (0 ≤ *x *≤ 0.1) nanocrystals have been successfully synthesized in aqueous media using a simple wet chemical precipitation technique. Crystallographic and morphological studies reveal the zinc blende nanostructures having average crystallite size approx. 3 nm. Energy resolved luminescence spectra report the quenching of host-related 425 nm emission and enhancement in luminescence quantum yield of dopant-related 599 nm emission, with the increasing concentration of Mn^2+ ^ions. Photo-catalytic activity of nanocrystals studied using MB dye as a test contaminant enhances with the addition of Mn^2+ ^ions in ZnS nanocrystals upto to optimal concentration (1 at. wt% of Zn^2+^), but the further increase of dopant concentration deteriorates photo-catalytic activity of Zn_1-*x*_Mn_*x*_S nanocrystals as the recombination of trapped carriers dominates the interfacial charge transfer at the higher dopant concentrations. This mechanistic information of photo-catalytic activity dependence on dopant concentration and luminescence quantum yield will significantly contribute to enhance the understanding of photo-initiated processes in semiconductor nanocrystals.

## Abbreviations

AAS: atomic absorption spectrometer; CB: conduction band; MB: methylene blue; PVP: polyvinyl pyrrolidone; ST: shallow trap; TEM: transmission electron microscope; VB: valence band; XRD: X-ray diffraction.

## Competing interests

The authors declare that they have no competing interests.

## Authors' contributions

MC: Performed experimental work. KS: Generated scientific idea, performed experimental work with first author and written the manuscript. ISS: Supported and participated in the technical work. HSB: Steer for generation of scientific idea and manuscript design.
